# Study on pharmacokinetic interactions between SHR2554 and itraconazole in healthy subjects: A single‐center, open‐label phase I trial

**DOI:** 10.1002/cam4.5028

**Published:** 2022-07-16

**Authors:** Kunhong Deng, Yi Zou, Chan Zou, Hong Wang, Yuxia Xiang, Xiaoyan Yang, Shuang Yang, Chang Cui, Guoping Yang, Jie Huang

**Affiliations:** ^1^ Center of Clinical Pharmacology The Third Xiangya Hospital, Central South University Changsha China; ^2^ School of Mathematics and Statistics Central South University Changsha China; ^3^ Research Center of Drug Clinical Evaluation of Central South University Changsha China; ^4^ Department of Pharmacy The Third Xiangya Hospital, Central South University Changsha China; ^5^ XiangYa School of Pharmaceutical Sciences Central South University Changsha China; ^6^ National‐Local Joint Engineering Laboratory of Drug Clinical Evaluation Technology Changsha China

**Keywords:** drug–drug interaction, EZH2 inhibitor, itraconazole, pharmacokinetics, SHR2554

## Abstract

**Background:**

SHR2554, a novel oral Enhancer of Zeste Homolog 2 inhibitor, shows broad‐spectrum anti‐tumor efficacy in preclinical studies. As SHR2554 is mainly metabolized by CYP3A4, it is helpful to conduct research on the effects of itraconazole, a strong inhibitor of CYP3A4‐metabolizing enzymes, on the pharmacokinetic characteristics and safety of SHR2554.

**Methods:**

We conducted a single‐center, open‐label pharmacokinetic study of itraconazole on SHR2554 in 18 healthy Chinese subjects. Subjects were orally administrated SHR2554 50 mg on Day 1, itraconazole 200 mg *Quaque* Die (QD) from Days 4 to 7, SHR2554 50 mg co‐administrated with itraconazole 200 mg on Day 8, and itraconazole 200 mg QD from Days 9 to 12. Then, 4 ml of venous blood was collected at predetermined time points. Plasma SHR2554 concentrations were analyzed using a validated high‐performance liquid chromatography tandem mass spectrometry method. Pharmacokinetic parameters were calculated using Phoenix WinNonlin v8.1.

**Results:**

The *C*_max_ of SHR2554 alone and in combination was 10.197 ± 7.0262 ng·ml^−1^ versus 70.538 ± 25.0219 ng·ml^−1^, AUC_0–∞_ was 50.99 ± 19.358 h·ng·ml^−1^ versus 641.53 ± 319.538 h·ng·ml^−1^, and AUC_0–*t*_ was 28.70 ± 18.913 h·ng·ml^−1^ versus 612.13 ± 315.720 h·ng·ml^−1^. Co‐administration of SHR2554 and itraconazole caused 7.73‐, 12.47‐, and 23.75‐fold adjusted geometric mean ratios increases in SHR2554 *C*_max_, AUC_0−∞_ and AUC_0−*t*_ respectively. The co‐administration regimen was well tolerated and had a good safety profile.

**Conclusions:**

Compared with a single dose of SHR2554 50 mg, the exposure of SHR2554 in vivo was significantly affected by the combined administration of itraconazole.

## Graphical abstract

Compared with a single dose of SHR2554 50 mg, the exposure of SHR2554 in vivo was significantly elevated by the combined administration of itraconazole. The co‐administration regimen was well tolerated and had a good safety profile.

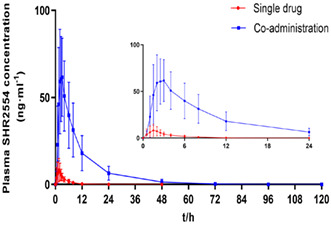

## 1 INTRODUCTION

Enhancer of Zeste Homolog 2 (EZH2) is the core component of the initiation complex Polycomb repressive complex 2 (PRC2)^1^ that can catalyze the trimethylation of lysine 27 of histone H3 (H3K27Me3) to inhibit target genes' expression.^2^ EZH2 functions as a key factor promoting tumor growth and metastasis in many malignancy models.^3^, ^4^ EZH2 plays a decisive role in immune cells such as T cells, natural killer cells, dendritic cells, and macrophages, which are essential components of the tumor microenvironment.^4^ Overexpression of EZH2 shows enhanced cell proliferation and oncogenic capacity that is usually associated with advanced stages of human cancer progression and poor prognosis.^5^ A growing body of research shows that inhibiting EZH2 with a small molecule inhibitor or gene knockout reduces cancer cell growth and tumor formation.^6^, ^7^, ^8^, ^9^, ^10^, ^11^ These observations have led to the development of specific EZH2 inhibitors. At present, many inhibitors specifically targeting EZH2 have been developed for the treatment of several cancers, such as relapsed or refractory follicular lymphoma,^12^ B‐cell lymphoma,^13^, ^14^, ^15^, ^16^, ^17^ advanced epithelioid sarcoma,^18^ advanced solid tumors,^17^, ^19^ advanced hematologic tumors,^19^ etc. Well‐studied inhibitors, including tazemetostat (EPZ6438),^12^, ^13^, ^15^, ^16^, ^17^, ^18^ CPI‐1205,^14^ CPI‐0209, GSK2816126,^19^ DS3210b, and PF‐06821497 are currently being tested in clinical trials.^20^

SHR2554 is a novel, highly effective, and selective oral EZH2 inhibitor developed by Jiangsu Hengrui Medicine Co., Ltd. It potently inhibits the activity of wild‐type and mutant EZH2 enzymes, thereby affecting intracellular H3K27Me3 levels in lymphoma cells and finally causing cell cycle arrest in the G1 phase. Moreover, SHR2554 induces early apoptosis of cancer cells and inhibits the growth of lymphoma both in vitro and in vivo. The combination of histone deacetylase inhibitor HBI8000 and EZH2 inhibitor SHR2554 exhibited dramatic antitumor activity in diffuse large B‐cell lymphoma (DLBCL).^21^ Therefore, SHR2554 could provide a potential therapeutic modality for the treatment of multiple malignant tumors.

The sponsor has initiated several SHR2554‐related clinical studies to assess the tolerability, safety, pharmacokinetics, and preliminary antitumor activity and food effects of SHR2554 (NCT03603951, NCT03741712, and NCT04335266). In the range of 50–400 mg Bis In Die (BID), the increased proportion of exposure was higher than that of the administered dose. As of January 3, 2020, two subjects in the 400 mg BID group developed grade three and four decreased platelet counts reported as serious adverse events (SAE)/dose‐limiting toxicity (DLT). And after the addition of the 300 and 350 mg dose groups, one subject in the 350 mg developed grade four neutropenia reported as SAE/DLT (NCT03603951). In addition, one subject developed grade two anemia, which was reported as SAE (NCT03741712). So, the recommended phase II dose of SHR2554 was 350 mg BID. The dose below 300 mg BID (including 300 mg BID) had a good safety profile, and no drug‐related SAE or DLT occurred. Our preclinical studies demonstrated that the oxidative metabolism of SHR2554 is mainly catalyzed by CYP3A4. In a clinal scenario, pharmacokinetics of SHR2554 would be significantly affected by its co‐administration with CYP3A4 inhibitors and/or inducers. Therefore, drug–drug interaction (DDI) clinical studies using strong index inhibitors and/or inducers of this metabolic enzyme should be conducted according to Chinese regulations.^22^ This study was designed to evaluate the pharmacokinetic effects of itraconazole, a potent CYP3A4 inhibitor recommended by DDI guidelines,^23^, ^24^ on SHR2554 and to establish the safety and tolerability of the combination medication in Chinese healthy adult subjects.

## 2 MATERIAL AND METHODS

### 2.1 Study design

This was a single‐center, open‐label, single‐dose, clinical trial to evaluate the effects of itraconazole on the pharmacokinetics and safety of SHR2554 in Chinese healthy adult subjects.

**FIGURE 1:**
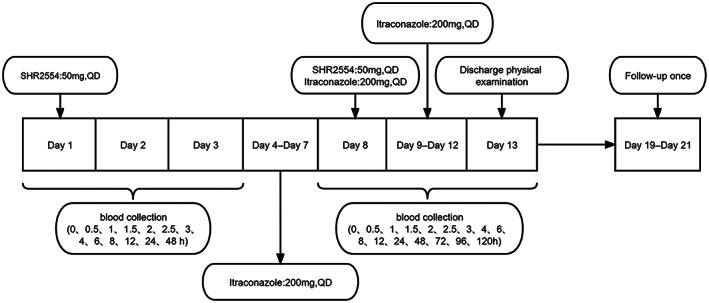
Trial process. QD, *Quaque* Die

Eighteen volunteers were recruited for the study. The subjects were orally administered SHR2554 at a 50 mg daily dose (Day 1); then, they were orally administered itraconazole 200 mg *Quaque Die* (QD) for 4 days (Days 4–7) followed by co‐administration of itraconazole 200 mg and SHR2554 50 mg (Day 8). They took itraconazole 200 mg QD for 4 days (Days 9–12). All subjects were fasted for at least 10 h, and they ate a standard meal in 30 min before drug administration. Water was forbidden within 1 h after drug administration, and the subjects were fasted for at least 4 h after drug administration. The clinical progress is shown in Figure 1.

Safety evaluation included adverse events, laboratory tests, physical examination, measurement of vital signs, 12‐lead electrocardiogram, etc. All abnormal and clinically significant laboratory results were followed up until they returned to normal.

### 2.2 Study population

Eligible subjects were recruited according to the listed criteria: healthy adults aged between 18 and 45 (inclusive), both men and women, with a male weight ≥50.0 kg and a female weight ≥45.0 kg, with a body mass index (BMI) of between 19.0 and 26.0 kg·m^−2^ (inclusive). The subjects were sexually inactive (abstinent or following contraceptive methods) from 2 weeks before the study (for female subjects only) to 3 months after the last administration (for both male and female subjects). The human chorionic gonadotrophin test of female subjects of child‐bearing potential was negative.

Subjects with clinically significant organ medical histories or hypersensitivity to the investigational drugs were excluded. In addition, laboratory screening tests (including prolonged QTc interval, left ventricular ejection fraction [LVEF] <50%, hepatitis B and hepatitis C positive, positive results on syphilis serological tests, and HIV positive) and abnormal vital signs were applicable to the exclusion criteria. Subjects who had taken drugs or food that affected the pharmacokinetics (PK) of the drug before the experiment were excluded. Additional exclusion criteria were being pregnant or breastfeeding, having a history of alcohol and drug abuse, donating blood and receiving blood transfusion, and being operated on within 6 months.

### 2.3 Study drug

Jiangsu Hengrui Pharmaceuticals Co., Ltd. produced and supplied SHR2554 tablets (specification: 50 mg/tablet; lot: 191205KG). Itraconazole capsules (specification: 100 mg/capsule; lot: 190606226) were produced by Xian Janssen Pharmaceutical Ltd. and supplied by Jiangsu Hengrui Pharmaceuticals Co., Ltd.

### 2.4 Pharmacokinetic assessments and bioanalysis

The samples of venous blood (4 ml) were collected into heparinized Vacutainer™ tubes and mixed gently. The first serial samples were collected from Day 1 within 60 min before drug administration and at 0.5, 1, 1.5, 2, 2.5, 3, 4, 6, 8, 12, 24, and 48 h after dosing. The second serial samples were collected from Day 8 within 60 min before administration and at 0.5, 1, 1.5, 2, 2.5, 3, 4, 6, 8, 12, 24, 48, 72, 96, and 120 h after dosing.

Thereafter, plasma was separated by centrifugation (2000*g* at 4°C for 10 min) within 1 h of sample collection and then stored at −80°C before analyses. The plasma concentrations of SHR2554 were determined by a validated method with sensitivity and specificity using high‐performance liquid chromatography–tandem mass spectrometry (HPLC‐MS/MS) by Shanghai InnoStar Bio‐tech Co. Ltd.

Blood samples were pretreated by protein precipitation with acetonitrile. Biological sample analysis was performed using SHR149633 (free base) as an internal standard. Separation of SHR2554 was carried out using a Waters ZORBAX Extend‐C18 column (2.1 × 50 mm, 1.8 μm), and analytes were detected on an AB SCIEX QTRAP 5500 mass spectrometer. The assay was linear over the range of 1–2000 ng·ml^−1^. The detection method for SHR2554 meets the requirements for precision and accuracy (≤15% [20% at the lower limit of quantification]). Plasma samples are stable under various conditions.

### 2.5 PK and statistical methods

The PK parameters were evaluated using Phoenix WinNonlin v8.1. Statistical analysis was performed with SAS v9.2 (SAS Institute). Descriptive statistics and tabulation of PK parameters for analytes were performed, and mean concentration‐time curves were plotted. After natural log transformation, the PK parameters were fitted with a mixed‐effects model. In the mixed‐effects model analysis, medication (itraconazole combined with SHR2554 or SHR2554 alone) was used as a fixed effect, and the participants in the model fitting were used as random effects. The corrected least‐square mean difference and 90% confidence interval (CI) between the administration of a single drug (SHR2554) alone and a combination drug (co‐administration of SHR2554 and itraconazole) were obtained. The corrected least‐square mean difference and its 90% confidence interval were converted to the adjusted geometric mean ratios (GMRs) (co‐administration of SHR2554 and itraconazole/SHR2554 alone) and its 90% confidence interval by taking the antilog.

## 3 RESULTS

### 3.1 Demographic characteristics

A total of 18 healthy subjects were enrolled in the study, of which 15 subjects completed it. However, two subjects withdrew because of adverse events (one on Day 5 due to rash and the other on Day 10 due to acute appendicitis) and one subject withdrew on Day 6 due to dysphagia (not related to the drug). The demographic data of the subjects are shown in Table 1.

**TABLE 1:** Baseline demographic characteristics of study volunteers (*n* = 18)

Characteristic, unit	Mean ± *SD*
Age, years	26.50 ± 5.92
Male, *n* (%)	16 (88.9)
Female, *n* (%)	2 (11.1)
Height, cm	166.61 ± 6.91
Weight, kg	61.23 ± 6.767
BMI, kg·m^−2^	22.047 ± 1.9446

Abbreviations: BMI, body mass index; *SD*, standard deviation.

### 3.2 Pharmacokinetics

**FIGURE 2:**
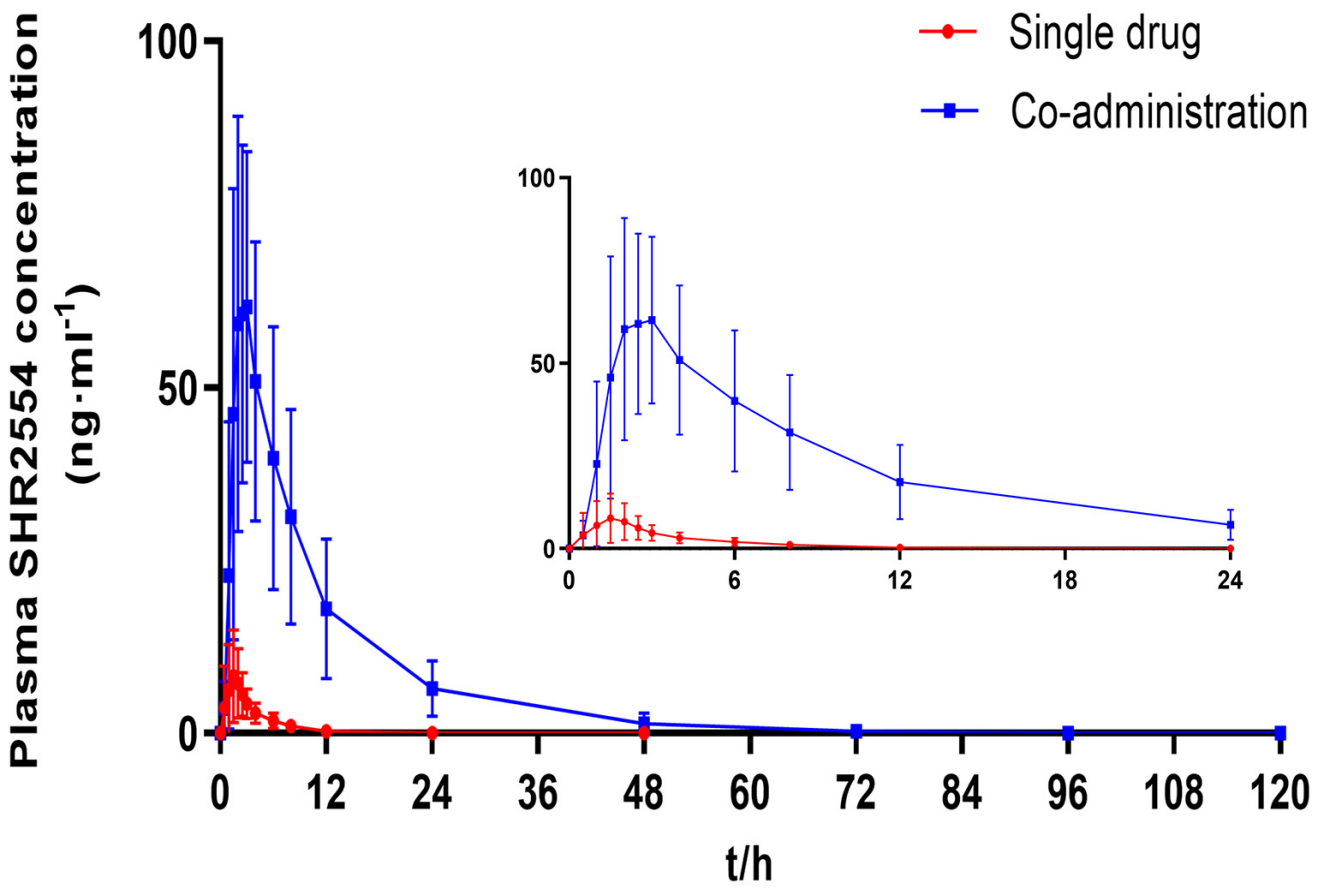
Mean ± *SD* (standard deviation) plasma SHR2554 concentration‐time curve of subjects under single drug and co‐administration with itraconazole

**FIGURE 3:**
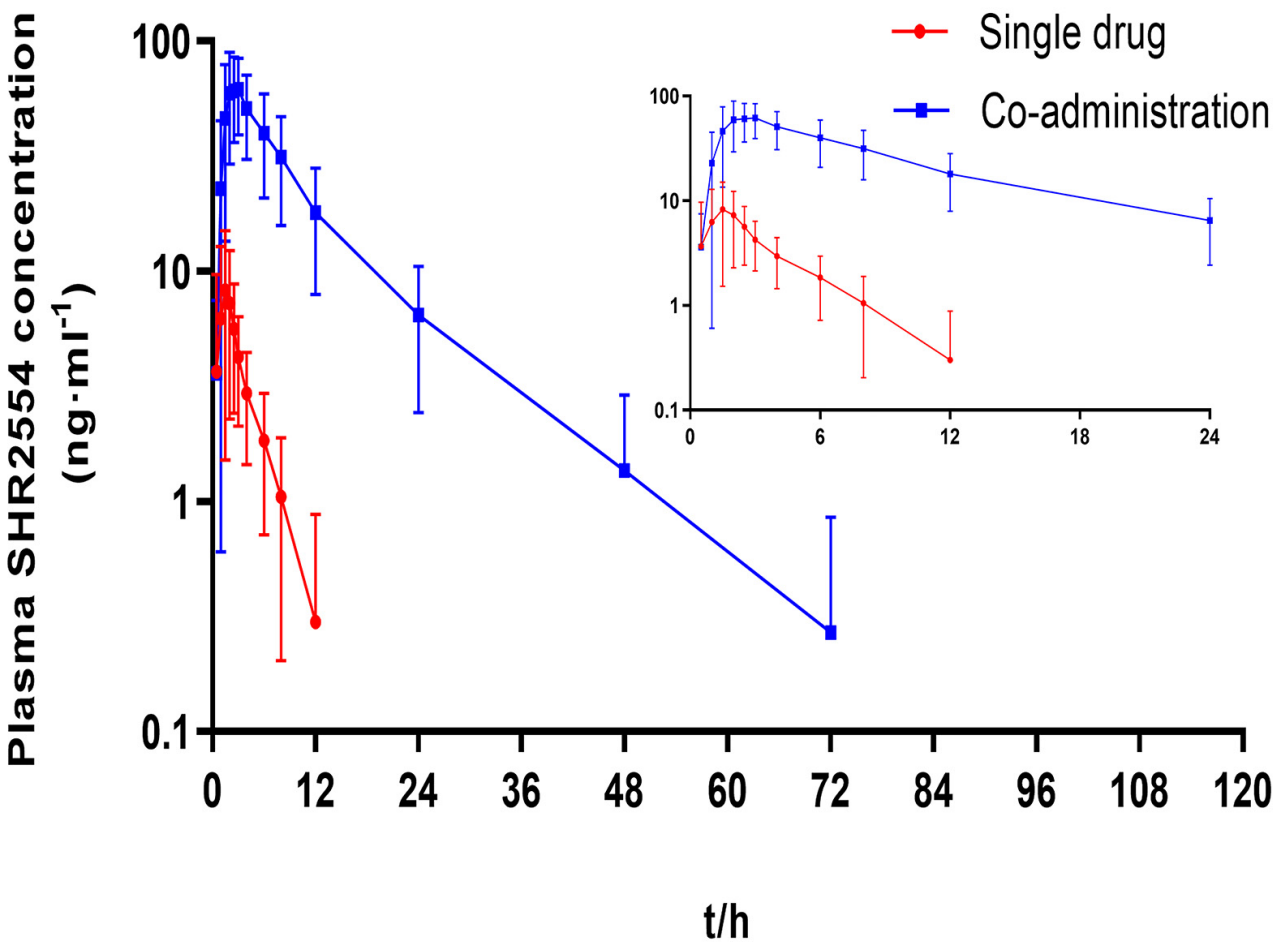
Mean ± *SD* (standard deviation) plasma SHR2554 concentration‐time curve of subjects under single drug and co‐administration with itraconazole (semi‐logarithmic)

**FIGURE 4:**
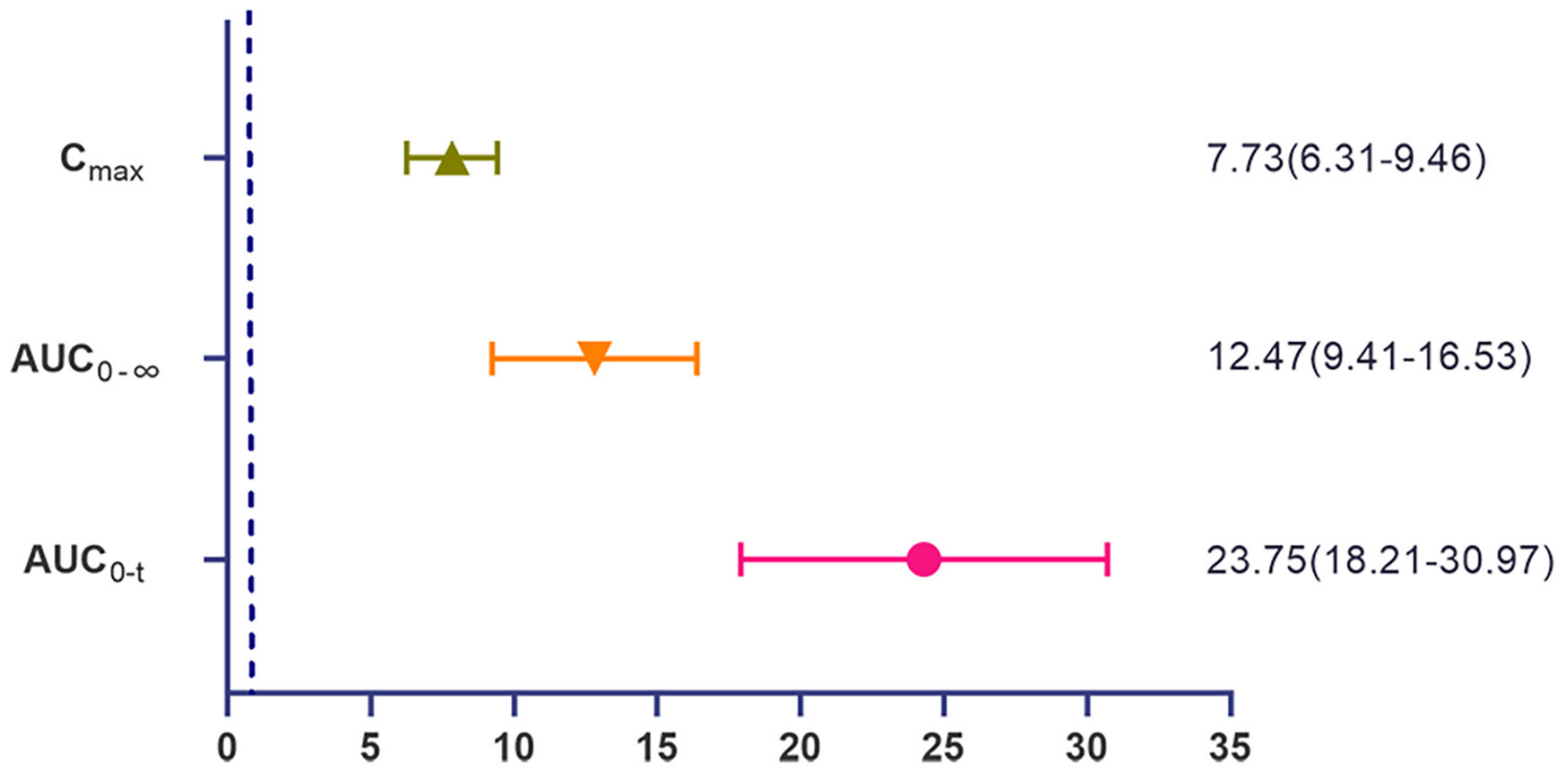
Forest plot with geometric least square mean ratios of co‐administration and single drug and 90% confidence interval. Transformed values on log scale (base e)

The mean plasma concentration‐time curves of SHR2554 under a single drug and co‐administration with itraconazole are presented in Figure 2 and the semi‐logarithmic curves are shown in Figure 3. In the single‐dose phase of SHR2554, the plasma concentration of all subjects was below lower limit of quantitation (LLOQ) at 24 h after administration. And, the plasma concentration of all subjects was below LLOQ at 96 h during SHR2554 combined with itraconazole administration. Moreover, the pharmacokinetic parameters of SHR2554 are shown in Table 2. The *C*_max_ of SHR2554 alone and in combination was 10.197 ± 7.0262 ng·ml^−1^ versus 70.538 ± 25.0219 ng·ml^−1^, AUC_0–∞_ was 50.99 ± 19.358 h·ng·ml^−1^ versus 641.53 ± 319.538 h·ng·ml^−1^, and AUC_0–*t*_ was 28.70 ± 18.913 h·ng·ml^−1^ versus 612.13 ± 315.720 h·ng·ml^−1^. The GMRs (90% CI) of the main PK parameters for SHR2554 are presented in Figure 4. Co‐administration of SHR2554 and itraconazole caused 7.73‐, 12.47‐, and 23.75‐fold GMRs increases in SHR2554 *C*_max_, AUC_0–∞_, and AUC_0–*t*_, respectively. The elimination half‐life of SHR2554 was significantly increased (3.287 ± 1.0636 h vs. 8.678 ± 3.6031 h), the apparent clearance rate was significantly reduced (2127.7 ± 1592.02 L·h^−1^ vs. 98.4 ± 46.96 L·h^−1^), and the body exposure was significantly increased.

**TABLE 2:** Pharmacokinetic parameters of SHR2554

PK parameters (unit)	Mean ± *SD* (CV%)	
	SHR2554	SHR2554 + itraconazole
*C*_max_ (ng·ml^−1^)	10.197 ± 7.0262 (68.91) [*N* = 18]	70.538 ± 25.0219 (35.47) [*N* = 16]
AUC_0–∞_ (h·ng·ml^−1^)	50.99 ± 19.358 (37.97) [*N* = 8]	641.53 ± 319.538 (49.81) [*N* = 15]
AUC_0–*t*_ (h·ng·ml^−1^)	28.70 ± 18.913 (65.90) [*N* = 18]	612.13 ± 315.720 (51.58) [*N* = 15]
CL/F (L·h^−1^)	2127.7 ± 1592.02 (74.82) [*N* = 18]	98.4 ± 46.96 (47.71) [*N* = 15]
*T*_max_ ^a^ (h)	1.472 ± 0.6286 (0.50, 3.00) [*N* = 18]	2.438 ± 0.7719 (1.50, 4.00) [*N* = 16]
*t*_1/2_ ^a^ (h)	3.287 ± 1.0636 (1.76, 5.94) [*N* = 18]	8.678 ± 3.6031 (5.15, 17.6) [*N* = 15]
*V*_*z*_/*F* (L)	8439.2 ± 3779.31 (44.78) [*N* = 18]	1060.7 ± 271.00 (25.55) [*N* = 15]
AUC_%Extrap^b^ (%)	14.498 ± 2.9731 (20.51) [*N* = 8]	5.379 ± 2.4084 (44.77) [*N* = 15]

Abbreviations: PK, pharmacokinetics; *SD*, standard deviation.

^a^ *T*_max_, *t*_1/2_, mean ± *SD* (minimum, maximum).

^b^ AUC_%Extrap, percentage of AUCINF_pred due to extrapolation from Tlast to infinity.

### 3.3 Safety

A total of 19 treatment emergent adverse events (TEAEs) occurred in 12 (12/18, 66.7%) subjects. All TEAEs are summarized in Table 3. Common TEAEs in this study included elevated blood triglycerides (27.8%), blood in the urine (11.1%), positive bacterial tests (11.1%), etc. Twelve subjects (12/18, 66.7%) had 16 SHR2554 drug‐related adverse events, and five (5/18, 27.8%) subjects had six itraconazole‐related adverse events. Table 4 shows the correlation between experimental drugs and TEAEs. Two cases of itraconazole‐related adverse events “increased blood triglycerides” started in the single‐dose phase of SHR2554 and ended after multiple dosing periods of itraconazole. The occurrence period of general adverse reactions was routinely defined as the time when adverse reactions began, so they were defined in the single‐dose phase of SHR2554 and related to SHR2554. But the events continued and ended after multiple administration period of itraconazole. Administration of itraconazole might also aggravate or prolong the duration of the adverse events of increased blood triglycerides. Therefore, these two adverse reactions were also determined to be possibly related to itraconazole.

**TABLE 3:** Summary of TEAEs

	SHR2554 (*N* = 18) (%)	Itraconazole (*N* = 18) (%)	SHR2554 + itraconazole (*N* = 16) (%)	Total (*N* = 18) (%)
Total	9 (50.0)	2 (11.1)	4 (25.0)	12 (66.7)
All kinds of inspection	8 (44.4)	1 (5.6)	3 (18.8)	0 (55.6)
Increased blood triglyceride	4 (22.2)	1 (5.6)	1 (6.3)	5 (27.8)
Blood in the urine	2 (11.1)	0	0	2 (11.1)
Positive bacterial tests	2 (11.1)	0	0	2 (11.1)
Positive urine leukocyte	0	0	1 (6.3)	1 (5.6)
Increased heart rate	1 (5.6)	0	0	1 (5.6)
Increased blood bilirubin	0	0	1 (6.3)	1 (5.6)
High blood uric acid	1 (5.6)	0	0	1 (5.6)
Kidney and urinary system diseases	2 (11.1)	0	0	2 (11.1)
Spermaturia	1 (5.6)	0	0	1 (5.6)
Hematuria	1 (5.6)	0	0	1 (5.6)
Infectious diseases	0	0	1 (6.3)	1 (5.6)
Appendicitis	0	0	1 (6.3)	1 (5.6)
Skin and subcutaneous tissue diseases	0	1 (5.6)	0	1 (5.6)
Rash	0	1 (5.6)	0	1 (5.6)
Cardiac organ diseases	1 (5.6)	0	0	1 (5.6)
Supraventricular extrasystole contraction	1 (5.6)	0	0	1 (5.6)

Abbreviation: TEAEs, treatment emergent adverse events.

**TABLE 4:** Correlation between experimental drugs and TEAEs

	SHR2554 (*N* = 18)		Itraconazole (*N* = 18)		SHR2554 + itraconazole (*N* = 16)		Total (*N* = 18)	
	Number of subjects (%)	Cases	Number of subjects (%)	Cases	Number of subjects (%)	Cases	Number of subjects (%)	Cases
TEAEs	9 (50.0)	13	2 (11.1)	2	4 (25.0)	4	12 (66.7)	19
SHR2554‐related TEAEs	9 (50.0)	12	1 (5.6)	1	3 (18.8)	3	12 (66.7)	16
Itraconazole‐related TEAEs	2 (11.1)	2	2 (11.1)	2	2 (12.5)	2	5 (27.8)	6

Abbreviation: TEAEs, treatment emergent adverse events.

Among the 19 instances (12 subjects) of TEAEs, according to the Common Terminology Criteria for Adverse Events (CTCAE), and 17 instances of TEAEs (11 subjects) were Grade 1, one instance (one subject) was Grade 2 (rash), and the last instance (one subject) was Grade 3 (acute appendicitis). No adverse events of grade four or above occurred, and all adverse events were recovered/resolved. Rash (Grade 2) and acute appendicitis (Grade 3) both led to subjects' dropping out of the trial. The rash occurred during multiple doses of itraconazole that may not be related to SHR2554 and may be related to itraconazole. Acute appendicitis occurred in the co‐administration phase, which may be related to SHR2554 but may not be related to itraconazole.

## 4 DISCUSSION

Based on the *Technical Guidelines for Drug Interaction Research (Trial)* published in China,^22^ if the contribution of a specific metabolic enzyme to the total elimination of the drug is ≥25%, based on the in vitro metabolic phenotype study and human pharmacokinetic study data, the enzyme can be considered to have a significant contribution to the elimination of the drug under investigation. At this time, DDI clinical studies should be conducted using strong index inhibitors and/or inducers of this metabolic enzyme. The Food and Drug Administration (FDA)^23^ and European Medicines Agency's^24^ guidelines also recommend that clinical DDI studies should start with a strong index inhibitor and a strong index inducer when evaluating the investigational drug as a substrate. Itraconazole is a potent CYP3A4 inhibitor and is recommended by guidelines for clinical DDI use. This study aimed to evaluate the effect of itraconazole on the PK effects of SHR2554 in healthy Chinese adult subjects. Our results demonstrated that co‐administration with itraconazole significantly increases the exposure of SHR2554, which caused an increase in the GMRs of *C*_max_, AUC_0–∞_, and AUC_0–*t*_ by 7.73‐, 12.47‐, and 23.75‐fold, respectively. This suggests that CYP3A4 plays a major role in the elimination of SHR2554 and that the drug–drug interactions of SHR2554 with other CYP3A4 inhibitors and/or inducers are worth noting.

In contrast with some interaction results between itraconazole and other drugs,^25^, ^26^, ^27^ itraconazole showed stronger inhibition of CYP3A4 enzyme, which significantly increased the exposure of SHR2554 in vivo and slowed the elimination. However, the degree of interaction in this study is smaller than in some drugs such as lovastatin.^28^ This may be related to the sensitivity of the target drug to itraconazole. The formation of the major metabolites of lovastatin is primarily catalyzed by CYP enzymes.^28^ Moreover, SHR2554 is also mainly metabolized by CYP3A4 but lower than lovastatin, while other drugs such as darolutamide^25^ are only partially metabolized by CYP3A4, and there are many other metabolic enzymes of other drugs. In addition, the dose of itraconazole also affects the magnitude of the interaction outcome, for example, there was a 15‐fold change in the AUC of lovastatin (acid) when the itraconazole dose was 100 mg,^28^ and a 20‐fold change in the AUC of lovastatin (acid) when the dose was 200 mg.^29^ These results are in good agreement with the knowledge of the biotransformation of drugs. Similarly, the experimental design of DDI, the number of days of itraconazole taken, the disease state of the subjects, etc. may affect the degree of the DDI effect.

A 7.73‐fold increase in *C*_max_ suggested that, except for the elimination process of SHR2554, the absorption process may also be affected after concomitant administration of itraconazole. This is consistent with our preclinical results, of which SHR2554 showed a low permeability in the Caco‐2 cell model, suggesting that efflux transporters permeability‐glycoprotein (P‐gp) may be involved.^30^ The efflux of antitumor drugs by P‐gp can lead to a decrease in the amount of drugs in tumor cells, thereby reducing the killing effect on tumor cells, a phenomenon called multidrug resistance (MDR). P‐gp can reduce the transmembrane absorption of the drug and reduce the blood drug concentration.^31^ In addition to being an inhibitor of CYP3A4, itraconazole is also an inhibitor of the efflux transporter p‐gp.^30^ The inhibitory effect of itraconazole on P‐gp reduces the efflux of the drug, increases the absorption, and improves the oral bioavailability of the drug,^32^ which is consistent with the increase in *C*_max_ and *V*_*z*_/*F*. Of course, itraconazole, as a strong inhibitor of CYP3A4, inhibits the metabolism of SHR2554, which is also part of the reason for the increase in *C*_max_ due to the slowed metabolism of SHR2554.

In the completed or ongoing clinical trials of SHR2554, the most common drug‐related TEAEs in hematology were decreased white blood cell count, thrombocytopenia, neutropenia, and hyperlipidemia, increased aspartate aminotransferase level, hypertension, elevated alanine aminotransferase, anemia, etc., whereas the non‐hematological common drug‐related TEAEs were mainly nausea and fatigue. In addition, some subjects experienced prolongation of the QT interval of the electrocardiogram, which was all clinically controllable. Some EZH2 inhibitors similar to SHR2554, such as tazemetostat,^12^ also had similar adverse effects such as thrombocytopenia, neutropenia, and anemia. Another analog, GSK2816126,^19^ had the most common adverse reactions such as fatigue and nausea. But in this trial, the adverse events related to SHR2554 were mainly increased blood triglycerides, blood in the urine, positive bacterial tests, and appendicitis. Some of them were new adverse reactions, which are different from those found in existing studies. Part of the reason may be that the effect of itraconazole on SHR2554 led to some new adverse reactions, but most of the possible reasons are due to individual differences in subjects and disease status. Factors such as experimental design, dosage, and days of administration may also lead to different adverse reactions. Although the drug is almost safe in the human study of SHR2554, the toxicity/adverse reactions of SHR2554 should be closely monitored, with special attention to hematological toxicity, cardiotoxicity, and gastrointestinal and muscular systems adverse reactions.

For itraconazole capsules, the drug instruction^33^ showed that itraconazole capsules should be taken with a full meal to ensure maximal absorption. For SHR2554, in a food effect study (NCT04335266), compared with SHR2554 100 mg administered on fasted condition, when healthy male volunteers took SHR2554 100 mg on fed condition, the GMRs of *C*_max_, AUC_0–*t*_ and AUC_0–∞_ were 0.83, 1.02 and 0.97, respectively; the 90% CIs of the GMRs of *C*_max_, AUC_0–*t*_ and AUC_0–∞_ were (0.64, 1.08), (0.86, 1.21), and (0.82, 1.15). The results showed that drug exposures were essentially the same in fasted and fed states. As itraconazole was recommended for postprandial administration, in order to keep the administration method consistent, both SHR2554 and itraconazole were administered in fed state uniformly.

The SHR2554 dose of 50 mg was tested in the DDI study. This was because the recommended phase II dose in previous clinical studies is 350 mg BID, and the safety below 300 mg BID was good. But considering that itraconazole is a strong inhibitor of CYP3A4 metabolizing enzymes, which increases the AUC of a sensitive index CYP substrate ≥5‐fold.^23^ Co‐administration with itraconazole may deeply decrease the metabolism of SHR2554 and increase the exposure of SHR2554. Therefore, this clinical study recommended a single dose of 50 mg, and the blood sample collection time for the combined administration of SHR2554 and itraconazole was longer than that for a single administration of SHR2554. This dose was selected for the study to minimize unnecessary drug exposure in healthy subjects. At the same time, compared to higher doses, 50 mg can observe a more obvious DDI effect as the competition for CYP3A4 is typically concentration dependent.^26^

Compared to EPZ‐6438 (tazemetostat), which is the first‐in‐class and the most widely researched oral EZH2 inhibitor, SHR2554 presented comparable anti‐tumor activity in vitro. Moreover, SHR2554 showed high sensitivity and was more effective in inhibiting cell H3K27Me3 and inhibiting cell proliferation, with a comparable or even better IC_50_ than EPZ‐6438.^21^ The tumor inhibition rate of SHR2554 was higher than that of the reference compound EPZ‐6438 at the same dose, but the serum and tumor systemic exposure were about one‐fifth of the systemic exposure of EPZ‐6438 at the same dose. In the CYP450 enzyme induction and enzyme inhibition tests of SHR2554, SHR2554 has a weak inhibitory effect on CYP3A4. On the contrary, only high concentration SHR2554 can induce the expression of the CYP3A4 enzyme. Therefore, these data suggest that SHR2554 is unlikely to precipitate clinically relevant PK drug–drug interactions due to CYP enzyme inhibition or induction of itself.

This study was performed in healthy subjects to minimize the effects of potential PK confounders such as concomitant medications, organ damage, and comorbidities.^34^ It proves that strong CYP3A4 inhibitors have a strong interaction with SHR2554, and the interaction study of CYP3A4 inducers (rifampin) with SHR2554 has also been completed (NCT04577885). In addition, the effects of other moderate inhibitors should be evaluated at a later stage to fully understand the DDI potential of the investigational drug. The effect of additional inhibitors can be assessed either in clinical interaction studies or through modeling and simulation methods, such as physiologically based pharmacokinetics (PBPK) modeling using validated perpetrator and substrate models. DDI studies can be used to inform potential future studies of concomitant use.^23^ Because SHR2554 is a novel, highly effective, and selective oral EZH2 inhibitor that may be frequently used in malignant tumors in the future, we hope that the results here will help researchers design and conduct future phase II/III studies and be good for clinical use and promotion of SHR2554.

## AUTHOR CONTRIBUTIONS

**Kunhong Deng:** Data acquisition, analysis and interpretation of the data, writing of the manuscript. **Yi Zou:** Data acquisition, analysis and interpretation of the data, writing of the manuscript. **Chan Zou:** Treatment of patients, data acquisition. **Hong Wang:** Data acquisition, analysis and interpretation of the data. **Yuxia Xiang:** Treatment of patients, data acquisition. **Xiaoyan Yang:** Treatment of patients, data acquisition. **Shuang Yang:** Treatment of patients, data acquisition. **Chang Cui:** Data acquisition, supervision and review. **Guoping Yang:** Research design, analysis and interpretation of the data, writing of the protocol. **Jie Huang:** Research design, analysis and interpretation of the data, writing of the protocol.

## FUNDING INFORMATION

This work was supported by the Key Research and Development Project of Hunan Province (2020SK2010), Hunan Provincial Natural Science Foundation of China (No. 2020JJ5852), National Natural Science Foundation of China (No. 81803639).

## CONFLICT OF INTEREST

The authors have no conflict of interest.

## ETHICS STATEMENT

The research protocol was approved by the ethics committee of the Third Xiangya Hospital of Central South University. We followed the International Ethical Guidelines for Human Biomedical Research (CIOMS, 2002), the current International Conference on Harmonization‐GCP, and the Declaration of Helsinki. Before performing the test procedure, the subject signed an informed consent.

## CLINICAL TRIAL REGISTRATION

The study was registered on ClinicalTrials.gov named “A Itraconazole Effect Study of SHR2554 on Healthy Chinese Adult Subjects” (NCT04627129).

## Data Availability

The data supporting this study were obtained from the corresponding authors, which are not publicly available due to privacy and ethical constraints.
